# Fast rhythm cycles as atomic fragments of cortical processing and learning

**DOI:** 10.1186/1471-2202-15-S1-P136

**Published:** 2014-07-21

**Authors:** Jenia Jitsev

**Affiliations:** 1 Functional Neural Circuits Group, Institute of Neuroscience and Medicine (INM-6) & Institute of Advanced Simulation (IAS-6), Forschungszentrum Juelich, 52425 Juelich, Germany

## 

Neuronal rhythms of different frequencies are ubiquitous in the brain activity. These rhythms are thought to be not just a mere epiphenomenon of neural dynamics, but to play an important role in information processing performed by the brain networks. However, the character of their functional involvement remains still largely elusive.

Fast brain rhythms in the gamma frequency range of 40-100 Hz, known to modulate both neuronal activity and synaptic plasticity, were often proposed to provide a reference frame for operations performed by cortical microcircuits [1,2]. More precisely, it was hypothesized that a flexible winner-take-all (WTA) computation is performed in a cycle of gamma oscillation by local fine-scale subnetworks that contain tightly coupled excitatory pyramidal neurons residing in cortical layer II-III. Such operation selects and amplifies a small population of pyramidal cells based on the incoming afferent input while suppressing the rest, rapidly generating a sparse code that represents the current stimulus in a course of a single gamma cycle. This hypothesis leaves open whether learning and memory trace formation as well may rely on fast rhythm cycles as discrete atomic fragments of ongoing processing.

We use here a hierarchical recurrent network that employs gamma cycle as an atomic fragment for unsupervised learning of object identity from natural image input [3]. Unsupervised learning runs on the top of a fast winner-take-all (WTA)-like computation performed within a single cycle of the ongoing fast rhythm. If given natural face images, the network is able to create memory traces containing reusable facial visual elements that are linked in associative, generative manner via simultaneously established bottom-up, lateral and top-down connectivity into a global person face identity. If a face image of a memorized person is presented, the network is able to rapidly recall its identity and gender in a single gamma cycle. The operation performed within a single cycle may be interpreted as a probabilistic inference of the latent causes that create the input and an estimation of the parameters of a mixture model with latent causes as its components. This computation has the character of an expectation-maximization procedure, where expectation part is carried out by WTA-like computation and maximization involves plasticity mechanisms that change synaptic strength and neural excitability over many repetitive cycles. Even if decoupled from external input, the network can self-generate activity in an off-line regime, replaying the memory content in a sequence of gamma cycles and improving its organization to generalize better over the novel face images not presented before once back in input-driven regime [4].

Thus, the presented network model provides interpretation of the gamma cycle as an elementary fragment of ongoing processing and learning, where each cycle embeds a winner-take-all-like computation that supports memory trace formation and memory trace maintenance in hierarchical recurrent network pathways of the cortex.
